# Respiratory Syncytial Virus Vaccination Strongly Induces RSVpreF-specific IgG and Neutralizing Activity in Immunocompromised Individuals

**DOI:** 10.1097/TXD.0000000000002001

**Published:** 2026-09-16

**Authors:** Saskia Bronder, Henning Gruell, Rebecca Urschel, Simone Lennartz, Dimitrij Tschausowsky, Amina Abu-Omar, Richard Radun, Danilo Fliser, Heinrike Wilkens, David Schmit, Martina Sester

**Affiliations:** 1 Department of Transplant and Infection Immunology, PharmaScienceHub (PSH), Saarland University, Homburg, Germany.; 2 Institute of Virology, Faculty of Medicine and University Hospital Cologne, University of Cologne, Cologne, Germany.; 3 Department of Internal Medicine IV, Saarland University Medical Centre, Campus Homburg, Homburg, Germany.; 4 Department of Internal Medicine V, Saarland University Medical Centre, Campus Homburg, Homburg, Germany.; 5 Center for Gender-specific Biology and Medicine (CGBM), Saarland University, Homburg, Germany.

## Abstract

**Background.:**

Protein-based respiratory syncytial virus (RSV) prefusion F (RSVpreF) vaccines show promising immunogenicity in immunocompromised individuals. We have recently shown that RSV-specific CD4 T-cell responses and IgG antibodies were significantly induced, but data on functional humoral responses as well as associations between cellular and humoral immunological endpoints remain limited. We therefore extended our previous studies and performed a head-to-head assessment of vaccine-specific RSVpreF IgG and neutralizing antibody activity across different immunocompromised populations, including kidney and lung transplant recipients, patients on hemodialysis, and patients with CKD.

**Methods.:**

Conformation-specific RSVpreF-specific IgG levels and RSV-neutralizing plasma activity were assessed before and 13–18 d after RSV vaccination in 61 patients with chronic kidney disease (CKD), 15 patients receiving hemodialysis, 46 kidney transplant (KTx) recipients, and 31 lung transplant (LuTx) recipients. RSVpreF-specific IgG was measured by ELISA and neutralizing activity using an RSV-pseudovirus assay. Furthermore, comprehensive correlation analyses were carried out to assess associations between vaccine-induced humoral immune parameters and cellular immunity.

**Results.:**

Vaccination significantly increased RSVpreF-specific IgG (*P* ≤ 0.0001) and neutralizing activity in all groups (*P* = 0.002 to *P* < 0.0001). Median fold increases in IgG were highest in patients with CKD (11.9-fold), followed by patients on hemodialysis (7.8-fold) and LuTx recipients (7.6-fold), and lowest in KTx-recipients (3.3-fold). Neutralizing activity showed a similar pattern, with fold increases of 10.9, 5.4, 7.5, and 2.6, respectively. MMF/MPA use and vaccination within the first year posttransplant were associated with reduced humoral responses. RSVpreF-specific IgG correlated strongly with neutralizing activity (r = 0.664, *P* < 0.0001), whereas correlations with CD4 T-cell responses were less pronounced.

**Conclusions.:**

RSV vaccination induces robust functional humoral immunity in all tested immunocompromised patient groups, but responses are lowest in kidney transplant recipients. Among transplant recipients, responses were reduced within the first year after transplantation and in patients on MMF/MPA, supporting the need for optimized vaccination strategies in these patient groups.

## Graphical abstract

**Figure:**
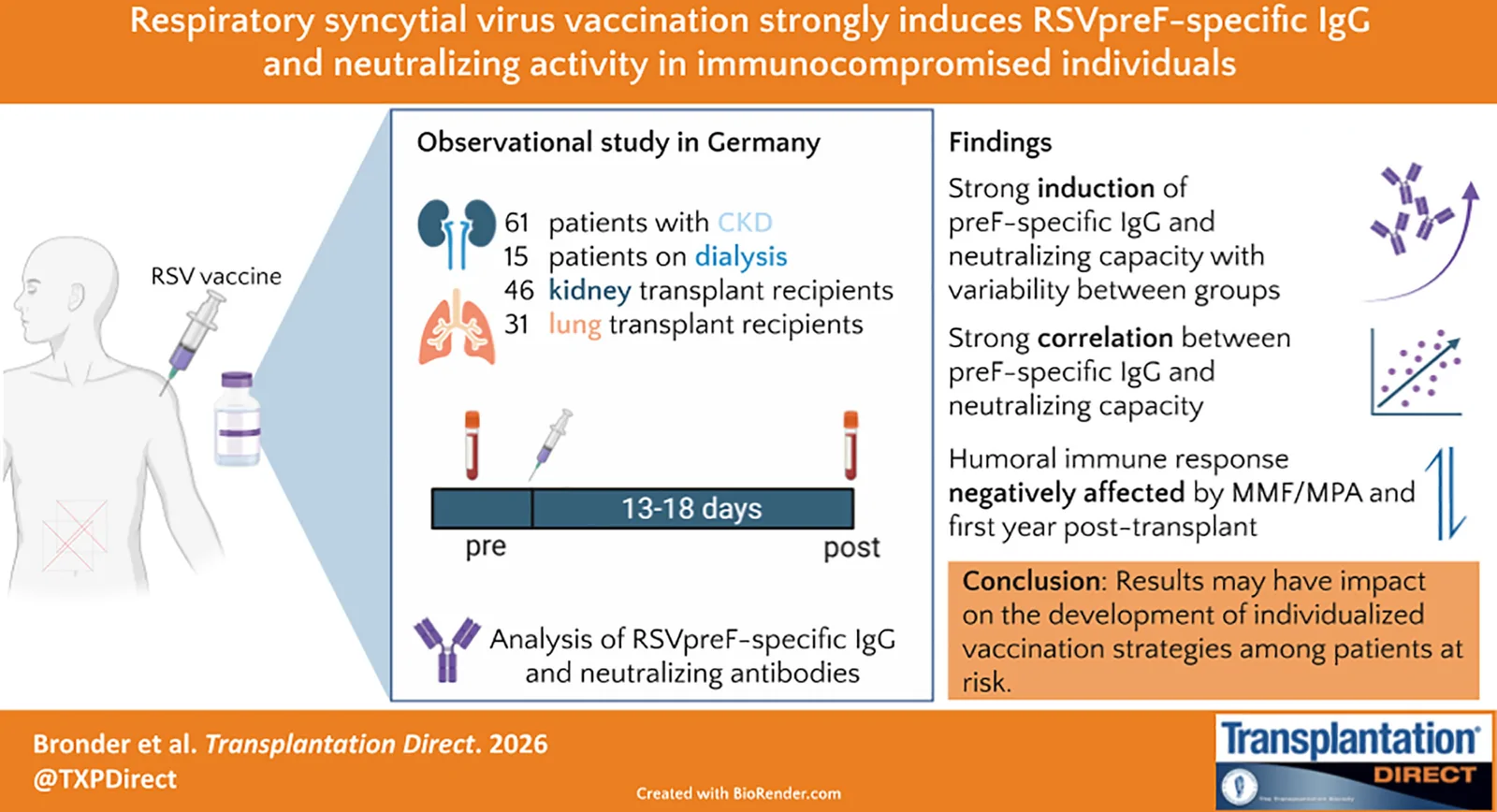

Respiratory syncytial virus (RSV) vaccines based on the prefusion F glycoprotein (preF) are now recommended for the elderly and immunocompromised individuals.^1,2^ In the pivotal trials, the mRNA-based vaccine mRNA-1345 and both protein-based vaccines—the nonadjuvanted, bivalent RSVpreF, and the adjuvanted, monovalent RSVpreF3-AS01_E_—achieved strong immunogenicity and efficacy in immunocompetent adults aged ≥60 y,^3-5^ but immunocompromised individuals were excluded from these trials. Recent studies have shown that protein-based^6-12^ or mRNA-based^13^ RSV vaccination also induced RSV-specific IgG and neutralizing antibodies in individuals with various immunocompromising conditions,^6,7^ solid organ transplant recipients,^8-10,13^ or allogeneic hematopoietic stem cell transplant recipients.^10-12^ However, only 4 studies simultaneously assessed both vaccine-induced IgG titers and neutralizing antibodies.^7,8,10,12^ Moreover, except for 1 study focusing on hematopoietic stem cell and lung transplant recipients,^10^ no head-to-head analyses of IgG specific to the prefusion conformation of the F protein (RSVpreF) and neutralizing antibodies among several groups of immunocompromised patients, and on their correlation with RSV-specific T-cell immunity are available. We have recently shown that a single dose of a protein-based RSV vaccine led to a robust induction of both RSV-specific CD4 T cells and IgG antibodies toward pan-RSV and F-protein among patients with chronic kidney disease (CKD) stages G3 to G5d^14^ as well as lung (LuTx) and kidney transplant (KTx) recipients.^15^ However, information on IgG antibodies toward the RSVpreF conformation of the vaccine and neutralizing antibody activity including their correlation with cellular immunity was lacking due to limited availability of suitable assays. By specifically quantifying antibodies against the prefusion F conformation used as the vaccine antigen, RSVpreF-based assays more accurately reflect vaccine-induced immunity than global or conformation-independent antibody assays. Moreover, information on neutralizing antibody levels enables a more comprehensive and clinically meaningful assessment of functional humoral immunity.

We therefore extended our previous studies^14,15^ and carried out a detailed head-to-head analyses of RSVpreF-specific IgG and neutralizing antibody activity before and after protein-based RSV vaccination in patients with CKD, on intermittent maintenance hemodialysis (HD), as well as patients after kidney and lung transplantation. Moreover, these data were correlated with previously described vaccine-induced IgG toward pan-RSV and RSV-F, and RSV-specific CD4 T cells.

## MATERIALS AND METHODS

### Study Design and Subjects

Plasma samples from our observational studies among patients with CKD stages G2 to G5, on intermittent maintenance hemodialysis (HD), after kidney (KTx) and lung (LuTx) transplantation^14,15^ were used in the present study. All patients were recruited from October 2024 to May 2025 at the Saarland University Medical Center in Homburg, Germany. Details of the recruitment strategy and study design were previously reported^14,15^ with further information given in the supplementary information. The study was approved by the ethics committee of the Ärztekammer des Saarlandes (reference 99/24), and written informed consent was obtained from all individuals.

### Determination of RSV-specific IgG Antibodies

IgG antibodies toward the RSVpreF-glycoprotein F0 were quantified in this study using an enzyme-linked immunosorbent assay (ELISA, Human Anti-RSV-F0 Antibody IgG Titer ELISA Assay Kit) according to the manufacturer’s instructions (ACROBiosystems AG, Basel, Switzerland). IgG levels were expressed in relative units (RU/mL). Samples were tested at a dilution of 1:3200 before vaccination and at a dilution of either 1:12800 or 1:102,400 after vaccination.

IgG antibodies toward pan-RSV (RU/mL) and the RSV-F protein (U/mL, independent of the conformation) were determined before using ELISA kits (anti-RSV-IgG, human anti-RSV fusion protein IgG) according to the manufacturer’s instructions (Euroimmun, Lübeck, Germany, Alpha Diagnostic International, TX) as previously described,^14,15^ and data were used for comparative analyses in this study.

### Quantification of Neutralizing Antibody Capacity

RSV Long strain neutralizing plasma activity was determined in a 293T cell-based pseudovirus assay using F and G protein-expressing pseudoviruses as previously described with modifications.^16^ Importantly, serum neutralization determined against such pseudoviruses has been demonstrated to show excellent correlation to serum neutralizing activity determined against full-length virus.^16^ HDM_RSV_Long_F and HDM_RSV_Long_G_31AACTdel plasmids were a gift from Jesse Bloom (Addgene #237349 and #237350), as were HDM-Hgpm2, HDM-Tat1b, pRC-CMV-Rev1b, and pHAGE-CMV-Luc2-IRES-ZsGreen-W plasmids.^17^ The generation of lentiviral pseudoviruses and determination of RSV-neutralizing plasma activity was performed as described in the supplementary methods.

### Quantification of RSV-specific CD4 T Cells

CD69^+^IFNγ^+^ RSV-specific CD4 T cells from the same patients were determined before from heparinized whole blood after a 6 h stimulation with overlapping peptides spanning selected proteins of RSV (2 µg/mL, PM-pan-RSVselect-1, jpt Berlin, Germany, with peptides derived from glycoprotein F0 (61%), matrix (11%), and nucleoprotein (11%)) as previously described,^14,15^ and data were used for comparative analyses in this study.

### Statistical Analysis

All statistical analyses were performed using GraphPad Prism 10.6.0 software (GraphPad, San Diego, CA) using two-tailed tests. Categorical analyses on sex were performed using X^2^ test. To compare paired data between 2 groups, Wilcoxon-signed rank test was used. Mann–Whitney and Kruskal–Wallis test followed by Dunn’s multiple comparison test were used to compare unpaired nonparametric data between groups. Correlations were analyzed using a correlation matrix according to Spearman. Multivariate linear regression analysis was performed from log(10) transformed values to test the effects of age, sex, time after transplantation, immunosuppression including MMF/MPA intake or a composite drug score, on vaccine-induced immunity. A 4-parameter variable slope regression curve was used to calculate ID_50_. In case datasets were missing, this is specified in each figure or table legend. A *P* value <0.05 was considered statistically significant.

## RESULTS

### Study Population

Samples from 61 patients with CKD stages G2a to G5a, 15 patients on intermittent maintenance hemodialysis (HD), 46 patients after kidney (KTx), and 31 patients after lung (LuTx) transplantation were included. Among patients with CKD, 1 was in KDIGO stage G2a, 18 in G3a, 21 in G3b, 17 in G4a, and 4 in G5a. All patients were tested before and after vaccination with a protein-based RSV vaccine with the study design shown in Figure S1 (SDC, https://links.lww.com/TXD/A895). The majority of patients received the adjuvanted vaccine RSVpreF3-AS01_E_ (all CKD, all HD, 45/46 KTx, and 21/31 LuTx). The nonadjuvanted RSVpreF was administered in 1 KTx and 10 LuTx recipients. Information on patient characteristics such as time after transplantation and immunosuppressive regimens including MMF/MPA are shown in Table S1 (SDC, https://links.lww.com/TXD/A895). Patients with CKD with and without kidney replacement therapy were significantly older than SOT recipients (*P* < 0.0001; Table S1, SDC, https://links.lww.com/TXD/A895). KTx and LuTx recipients received their transplant at a median of 5.9 (IQR 9.9) and 5.3 (IQR 7.5) y ago, respectively. Ten out of 61 patients with CKD, 1/15 patient on dialysis and all SOT recipients received immunosuppressive therapy. Among SOT recipients, the majority (LuTx, 27/31; KTx, 35/46) received an immunosuppressive triple-drug regimen including glucocorticoids, an antimetabolite, and a calcineurin inhibitor.

### Lower Levels of RSVpreF-specific IgG and Neutralizing Activity in Kidney Transplant Recipients

**FIGURE 1.**
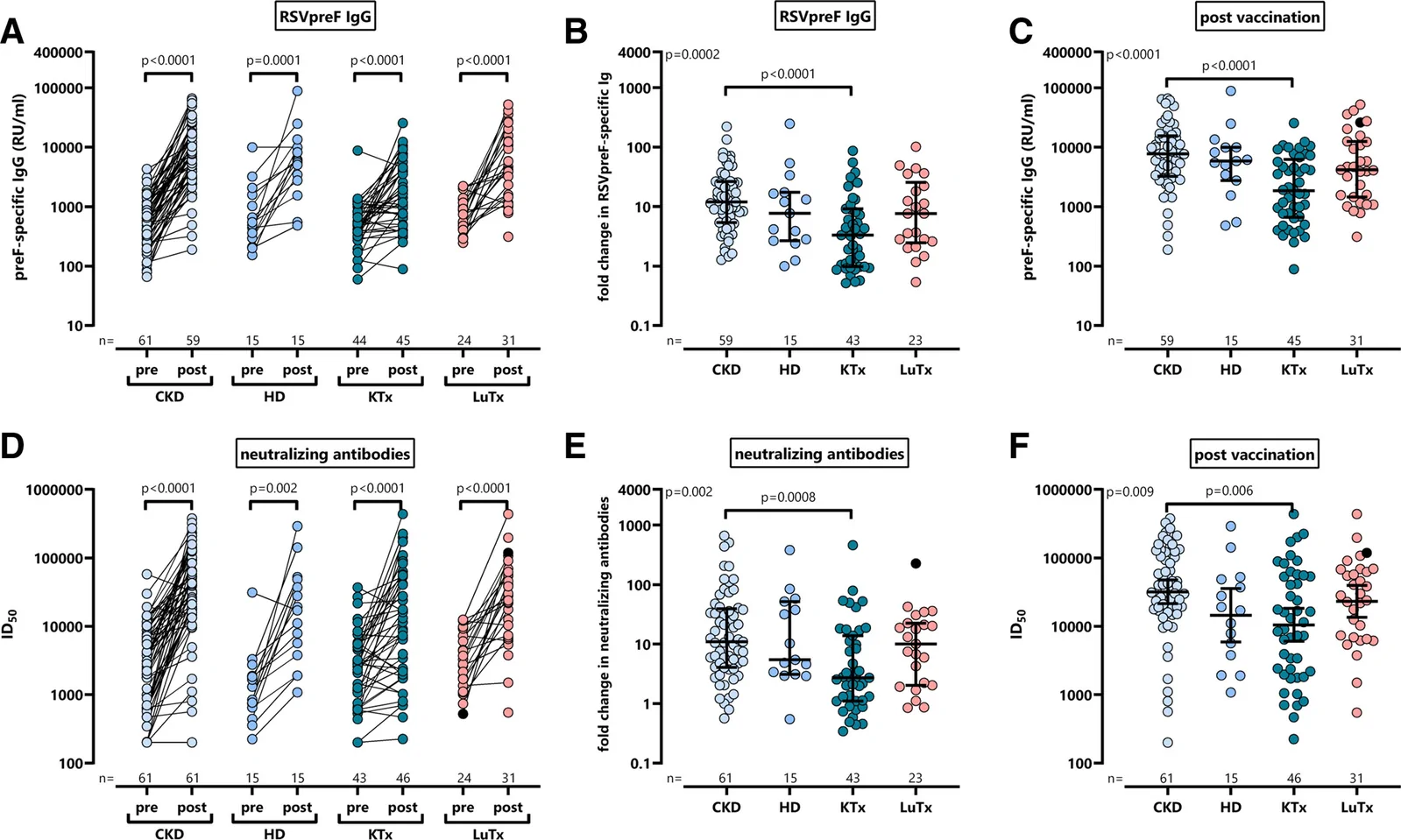
Vaccine-induced RSVpreF-specific IgG antibodies and neutralizing activity. Levels of (A) preF-specific IgG and (D) RSV-specific neutralizing antibody activity were analyzed before and after RSV vaccination in patients with CKD, patients on intermittent maintenance HD, KTx, and LuTx recipients. The fold change in (B) preF-specific IgG and (E) RSV-specific neutralizing antibody activity were compared between the patient groups. Comparison of vaccine-induced (C) preF-specific IgG and (F) RSV-specific neutralizing antibody activity in patients with CKD, patients on HD, KTx, and LuTx recipients. Bars represent median titers with interquartile ranges for preF-specific IgG and fold increases, and geometric mean ID_50_ with 95% CI for neutralizing antibodies. One LuTx recipient, marked with a black dot, had an RSV infection between vaccination and sampling after vaccination. Data from this patient are displayed but were excluded from statistical analysis, as the induction observed after vaccination may be confounded by the infectious episode. Differences were calculated using Wilcoxon-signed rank test (before/after) or Kruskal–Wallis with Dunn’s posttest for group comparisons. Median values (IQR) or geometric mean ID_50_ with 95% CI before and after vaccination are summarized for the four groups in the Table S2, SDC, https://links.lww.com/TXD/A895. CKD, chronic kidney disease; HD, hemodialysis; ID_50_, half-maximal inhibitory concentration; Ig, immunoglobulin; KTx, kidney transplant recipients; LuTx, lung transplant recipients; preF, prefusion F; RSV, respiratory syncytial virus; RU, relative unit.

IgG toward the RSVpreF-glycoprotein F0 (preF) were detectable in all groups prior to vaccination, with no differences between the groups (*P* = 0.198; Table S2, SDC, https://links.lww.com/TXD/A895). Vaccination led to a significant induction of RSVpreF-specific IgG in all groups (Figure 1A), although the median fold change before and after vaccination differed between the groups (*P* = 0.0002; Figure 1B). The median fold change was highest in patients with CKD (11.9 [IQR 20.9] fold), followed by patients on dialysis (7.8 [IQR 14.7] fold) and LuTx recipients (7.6 [IQR 23.1] fold) and was lowest in KTx recipients (3.3 [IQR 8.2] fold; Figure 1B; Table S2, SDC, https://links.lww.com/TXD/A895). Consequently, median levels of RSVpreF-specific antibodies after vaccination showed significant differences between the groups (*P* < 0.0001), with highest levels in patients with CKD (7754 [IQR 12 289] RU/mL) followed by patients on dialysis (5863 [IQR 7179] RU/mL) and LuTx recipients (4153 [IQR 11 072] RU/mL) and lowest levels in KTx recipients (1843 [IQR 5552.3] RU/mL; Figure 1C; Table S2, SDC, https://links.lww.com/TXD/A895).

As shown by a RSV pseudovirus-based neutralization assay, baseline neutralizing plasma activity (geometric mean ID_50_) did not differ between the groups (*P* = 0.074; Table S2, SDC, https://links.lww.com/TXD/A895). Neutralizing activity significantly increased in all groups after vaccination (Figure 1D, from *P* = 0.002 to *P* < 0.0001). Again, as with preF-specific IgG antibodies, the fold increase in neutralizing activity from pre-to postvaccination as well as titers after vaccination differed between the groups (Figure 1E and F; *P* = 0.002 and *P* = 0.009, respectively). The increase and geometric mean postvaccine titers were highest in patients with CKD (10.9-fold, ID_50_ 31953 [IQR 26 336]), followed by patients on dialysis (5.4-fold, ID_50_ 14488 [IQR 29 681]) and LuTx recipients (7.5-fold, ID_50_ 21983 [IQR 24 915]), and lowest in KTx recipients (2.6-fold, ID_50_ 10497 [IQR 12 352]; Table S2, SDC, https://links.lww.com/TXD/A895; Figure 1E and F). When comparing vaccine-induced RSVpreF-specific IgG and neutralizing activity between LuTx recipients receiving the nonadjuvanted RSVpreF (n = 10) or the adjuvanted RSVpreF3-AS01_E_ vaccine (n = 21), immune responses were comparable regardless of the vaccine administered (Figure S2, SDC, https://links.lww.com/TXD/A895).

Finally, multivariable linear regression analysis adjusted for age and sex was performed, which confirmed differences in vaccine-induced RSVpreF-specific IgG levels and neutralizing activity between the 4 groups, whereas age and sex had no confounding effects on our results (Table S3, SDC, https://links.lww.com/TXD/A895). Among SOT recipients, vaccination in the first year after transplantation and MMF/MPA intake was associated with lower vaccine-induced RSVpreF-IgG levels and plasma neutralizing activity (Table 1, model 1), whereas a composite drug score had no confounding effect on the 2 parameters (Table 1, model 2).

**TABLE 1.** Multivariable regression analyses of RSVpreF-specific IgG and neutralizing activity between kidney and lung transplant recipients

Dependent variables^*a*^		IgG RSVpreF		Neutralizing activity	
Model 1		Estimate (95% CI)	*P*	Estimate (95% CI)	*P*
SOT group	KTx (ref)	1		1	
	LuTx	0.203 (−0.059 to 0.466)	0.126	0.126 (−0.221 to 0.472)	0.472
First year after transplantation	No (ref)	1		1	
	yes	−0.372 (−0.692 to −0.053)	**0.023**	−0.442 (−0.865 to 0.019)	**0.041**
MMF/MPA	no (ref)	1		1	
	yes	−0.511 (−0.807 to −0.216)	**0.001**	−0.635 (−1.026 to −0.244)	**0.002**
**Model 2**		**Estimate (95% CI**)	***P***	**Estimate (95% CI**)	***P***
SOT group	KTx (ref)	1		1	
	LuTx	0.383 (0.102 to 0.665)	**0.008**	0.308 (−0.055 to 0.670)	0.095
First year after transplantation	no (ref)	1		1	
	yes	−0.278 (−0.678 to 0.123)	0.172	−0.355 (−0.861 to 0.151)	0.166
Immunosuppression drug score^*b*^	no	−0.092 (−0.418 to 0.234)	0.965	0.009 (−0.415 to 0.434)	0.965

*P* values of multivariable linear regression analyses with log(10)-transformed values after vaccination using 2 different models; significant *P*-values are marked in bold font.

^*a*^Parameters refer to RSV-specific humoral immune responses (preF-specific IgG and neutralizing activity) after vaccination with KTx recipients, >1 y after transplantation, and no MMF/MPA intake as a reference for categorical parameters.

^*b*^The composite immunosuppression drug score was determined as described before.^18^

One LuTx recipient had an RSV infection between vaccination and sampling after vaccination and was therefore excluded from statistical analysis.

CI, confidence interval; Ig, immunoglobulin; KTx, kidney transplant recipient; LuTx, lung transplant; MMF, mycophenolate mofetil; MPA, mycophenolic acid; preF, prefusion F; RSV, respiratory syncytial virus; SOT, solid organ transplant.

### Comparison of Vaccine-induced RSV-specific Humoral and Cellular Immune Responses

We have previously shown that IgG antibodies toward pan-RSV and the conformation-independent F-protein, as well as RSV-specific CD4 T cells were also shown to be significantly induced in all patient groups.^14,15^ When comparing all parameters, the median fold change in RSVpreF-specific IgG and neutralizing activity from pre- to postvaccination determined in this study was at least twice as high and up to 5 times higher than previously determined for pan-RSV- and F-specific IgG, and RSV-specific CD4 T cells (Table S4, SDC, https://links.lww.com/TXD/A895).

**FIGURE 2.**
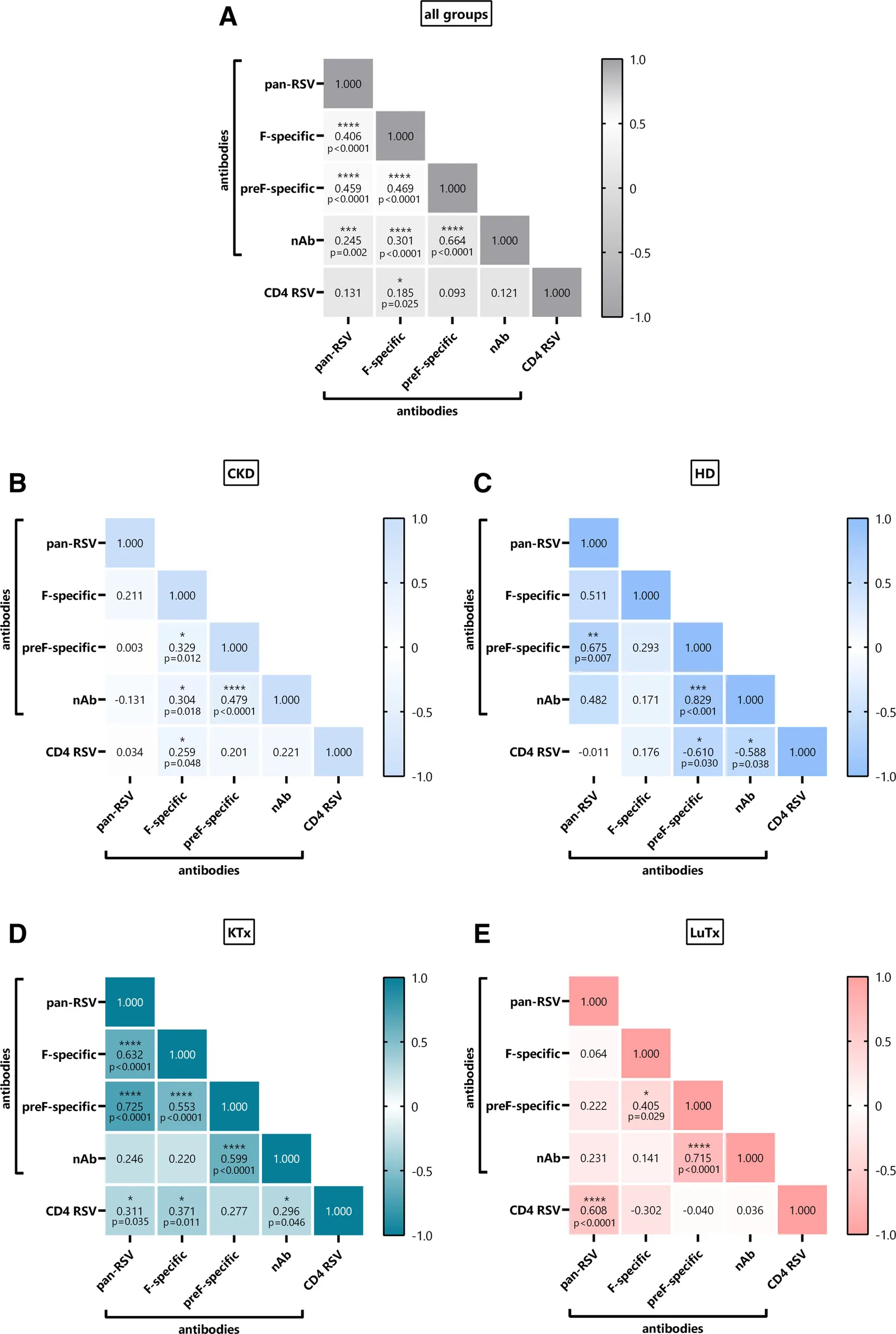
Correlation between vaccine-induced humoral and cellular immunity. Correlation matrix between vaccine-induced RSV-specific CD4 T cells with IgG levels toward pan-RSV, F-protein and preF-protein as well as neutralizing antibody activity among (A) all patient groups, and after stratification in (B) patients with CKD, (C) patients on HD, (D) KTx, and (E) LuTx recipients. Correlation coefficients were calculated according to 2-tailed Spearman and displayed using a color code, and *P* values (including stars denoting levels of statistical significance) are indicated. The LuTx recipient who had an RSV infection between vaccination and sampling after vaccination was excluded from this analysis. Data on RSV-specific CD4 T cells, and on IgG levels toward pan-RSV, and RSV-F-protein (conformation-independent) were derived from our previous studies,^14,15^ and used here for comparative analysis with IgG levels toward RSV-preF and neutralizing antibody activity. CKD, chronic kidney disease; HD, hemodialysis; Ig, immunoglobulin; KTx, kidney transplant recipient; LuTx, lung transplant recipient; nAb, neutralizing antibodies; preF, prefusion F; RSV, respiratory syncytial virus; SEB, *Staphylococcus aureus* Enterotoxin B.

Finally, we analyzed correlation patterns among all tested vaccine-induced humoral and cellular parameters (Figure 2). When data for all groups were combined, all humoral immune parameters showed a strong correlation (Figure 2A). After stratification into the 4 patient groups, the correlation between RSVpreF-specific IgG levels and neutralizing activity remained significant throughout the 4 groups, whereas all other correlations were less pronounced and showed differences among the 4 groups (Figure 2B–E).

## DISCUSSION

We have recently shown that administration of a single dose of a protein-based RSVpreF-based vaccine led to a pronounced induction of RSV-specific CD4 T cells in patients with CKD and SOT recipients.^14,15^ All patients also showed a significant induction in RSV-specific antibodies, although these analyses were restricted to antibodies toward pan-RSV and the F-protein tested in a conformation-independent assay. We now extended our results by more specific antibody data on IgG toward the RSVpreF-conformation contained in the vaccine and by functional data on plasma neutralization using an RSV-pseudovirus assay that primarily determines anti-RSV F antibody-mediated activity.^16^ These data reveal that the magnitude of induction of both RSVpreF-specific IgG and neutralizing activity was more pronounced than what was previously reported for pan-RSV or RSV-F-specific antibodies. Vaccine-induced RSVpreF-specific IgG and neutralizing capacity were comparable between patients with CKD, on dialysis and LuTx recipients, whereas KTx recipients showed the lowest titers. In line with our previous observations, multivariate analyses showed that titers for both parameters after vaccination were adversely affected by being in the first year after transplantation and MMF/MPA intake. Overall, RSVpreF-specific IgG and neutralizing activity strongly correlated in all patient groups, whereas correlations between antibodies and CD4 T cells were less pronounced. Our data are timely and broaden our knowledge on functional RSV vaccine immunogenicity at a time when RSV vaccination is being implemented in routine clinical care.

Consistent with the seasonal epidemiology of RSV infections,^19^ all individuals had pan-RSV-specific antibodies prior to vaccination.^14,15^ We now show that this also held true for the vaccine-specific RSVpreF-specific IgG antibodies and neutralizing activity at baseline. In line with the prefusion conformation of the F protein used as vaccine antigen, this preexisting RSVpreF-specific immunity and neutraling activity was boosted to a much greater extent than IgG antibodies toward pan-RSV and the conformation-independent RSV-F-protein, indicating an underestimation of the humoral immunogenicity in immunocompromised patients described previously,^14,15^ which also may extend to other studies that have used the less specific conformation-independent RSV-F IgG assay.^9^ Furthermore, despite some variability in the correlation depending on the tested humoral parameter and patient groups, the strongest correlation was observed between vaccine-induced RSVpreF-specific IgG and neutralizing antibody activity throughout all patient groups, which is consistent with the neutralizing epitopes specifically exposed on RSVpreF. Correlations between cellular and humoral immunity after pre-F vaccination were not found in all patient groups despite similarities in antigen specificities, indicating that the lack of association in some patient groups seems to reflect differences in the specificity of baseline immunity and distinct regulation and maintenance of cellular and humoral immune responses following RSV antigen exposure.

Our observations of a pronounced induction of neutralizing activity in all patient groups is in line with studies among lung transplant recipients^8,10^ and mixed cohorts of immunocompromised patients.^6,7,20,21^ Together these comparative data confirm that a protein-based RSV vaccination provides robust immune responses across vulnerable patient populations but also indicate differences in the magnitude among solid organ transplant recipients, patients with CKD, and patients undergoing hemodialysis, which may have potential implications for effectiveness of this vaccine in vulnerable patient groups.

When evaluating the magnitude of vaccine-induced immunity, the pivotal trial has shown that RSV-specific neutralizing activity and RSVpreF-specific IgG were induced approximately 10-fold and 13-fold, respectively, in elderly after RSVpreF3-AS01_E_ vaccination.^3^ Although interpretation of differences across different studies is limited by heterogenicity in assay methodologies, it is interesting to note that we observed an induction of a comparable magnitude in patients with CKD stages G2 to G5. In contrast, the immune response was less pronounced in patients undergoing intermittent hemodialysis and SOT recipients, which is in line with the fact that these patient groups have higher immunological impairments due to uremic immunodeficiency^22^ or immunosuppressive drugs. Both have previously been associated with impaired immune responses after various vaccinations such as SARS-CoV-2,^23,24^ influenza,^25^ pneumococcus, or tetanus.^26,27^ Consistent with our observations, a study among a mixed population of patients aged ≥60 y mainly including SOT recipients, patients with autoimmune diseases, and patients on intermittent hemodialysis, had a 7.9-fold and 8.5-fold increase of neutralizing antibodies toward RSV-A and RSV-B, respectively.^6^ In contrast, the 7.6-fold increase in RSVpreF-specific IgG levels and the 7.5-fold increase in neutralizing antibody titers that we observed in lung transplant recipients were somewhat higher than those reported in 2 previous studies.^8,10^ Although the majority of our patients had received the adjuvanted vaccine, we did not find any evidence for a different immunogenicity of the nonadjuvanted vaccine.

Among SOT recipients, we found that MMF intake and being in the first year after transplantation were confounders adversely affecting both RSVpreF-specific IgG and neutralizing activity, whereas overall immunosuppressive drug dosage did not seem to have an effect. This is in line with other studies that had included SOT recipients after vaccination toward RSV^6^ or SARS-CoV-2.^28-30^ Despite some subtle numerical differences, RSVpreF-specific IgG levels and neutralizing activity did not differ between kidney and lung transplant recipients. Interestingly, while MMF did not have any measurable adverse effect on vaccine-induced cellular immunity,^15^ patients in the first year after transplantation showed both a lower humoral and cellular immune response.^15^ Based on vaccine immunogenicity, these findings suggest that vaccination—when affordable—may be postponed to a time later after transplantation where the dosage of immunosuppressive drugs can be reduced or modulated. However, timing of vaccination will also have to include data on efficacy. Moreover, as patients in the first year after transplantation are at an increased risk of severe RSV infection due to higher levels of immunosuppression, timing of vaccination also necessitates a careful risk–benefit assessment depending on individual risk and seasonality.

A strength of our study is the integrated analysis of RSVpreF-specific and neutralizing antibodies, together with prior evaluations of previously determined parameters on RSV-specific humoral and cellular immune responses. Given the considerable lack of knowledge on RSV-vaccine-induced immunity in immunocompromised patients, this approach allowed for a comprehensive characterization of the immunogenicity across various patient groups including patients with CKD, on intermittent maintenance hemodialysis, after kidney and lung transplantation. Despite the strength of examining different patient groups with varying levels of immune dysfunction, age-matching was challenging between patient groups due to differences in RSV-vaccine recommendations between patient groups at risk. In addition, the study is limited by the fact that no age-matched immunocompetent individuals were included and our analysis of neutralizing activity was restricted to RSV subtype A, as the majority of patients had received the adjuvanted RSV-vaccine containing RSV-A only. However, as expected from the high sequence conservation of the RSV F protein, previous analyses in older adults consistently demonstrated that the protein-based RSV vaccines increase serum neutralization against both RSV subtypes A and B to a highly similar extent.^3,31,32^ Another limitation is the lack of systematic screening for RSV infections using polymerase chain reaction in the season prior to vaccination and throughout the study period. Moreover, no long-term follow-up data are available for our patients. However, the study is ongoing and includes analyses on clinical events such as the incidence of infectious episodes and on the durability of vaccine-induced immunogenicity, which may provide a basis for individualized recommendations on revaccination.

In conclusion, a single dose of a protein-based RSV vaccine led to a more pronounced induction of both RSVpreF-specific and RSV-specific neutralizing antibodies than was previously estimated based on assays that were less specific towards the prefusion F conformation of the vaccine. Our study also revealed differences in immunogenicity between individual patient groups, with MMF intake as confounder for humoral immunity and early time after transplantation as confounders for both humoral and cellular immunity. Together with emerging data on vaccine-effectiveness that require larger sample sizes, and more detailed knowledge on the role of humoral and cellular immunity for protection from RSV-infection and disease, these data may inform the development of individualized vaccination strategies.

## ACKNOWLEDGMENTS

The authors thank Candida Guckelmus for excellent technical assistance, and Susanne Brehmer, Inna Vallar, Fabio Lizzi, and the team of the Saarland University Transplant center for their support in enrolling participants. H.G. is supported by the Else Kröner-Fresenius-Stiftung. The authors also thank all participants to this study who contributed to the gain in knowledge from this project. Drawings in figures were in part generated by BioRender.

## Supplementary Material


