# Supplementary material for: Respiratory Syncytial Virus Vaccination Strongly Induces RSVpreF-specific IgG and Neutralizing Activity in Immunocompromised Individuals
Source: Transplant Direct. 2026 Sep 16;12(10):e2001. doi: 10.1097/TXD.0000000000002001 (PMC13593151; doi:10.1097/TXD.0000000000002001)
Supplement: Supplementary file 1 [file txd-12-e2001-s001.pdf]

Respiratory syncytial virus vaccination strongly induces RSVpreF-specific IgG and neutralizing activity in immunocompromised individuals

Saskia Bronder, MSc, Henning Gruell, MD, Rebecca Urschel, MSc, Simone Lennartz, MD, Dimitrij Tschausowsky, MD, Amina Abu-Omar, Richard Radun, MD, Danilo Fliser, MD, Heinrike Wilkens, MD, David Schmit, MD, Martina Sester, PhD

Supplementary Information

The supplement contains supplementary methods, 2 supplementary figures and 4 supplementary tables.

Supplementary methods

Study design and subjects

Details of the recruitment strategy and study design were previously reported[1,2]. In brief, all patients willing to participate were consecutively enrolled prior to administration of a single dose of either the protein-based adjuvanted vaccine RSVpreF3-AS01$_E$ ("Arexvy", GSK) or the nonadjuvanted RSVpreF ("Abrysvo", Pfizer) based on standard German recommendations[3]. Heparinized blood samples were collected before and after vaccination (anticipated after 13-18 days, with a longer time period tolerated for the post-vaccination visit, if patients had a longer distance from their home to the transplant center). Two weeks after vaccination were chosen in a variety of our vaccine studies as a meaningful timepoint capturing the induction of both antibodies and T cells[1,4-7]. Information on immunosuppressive drug regimens was collected for each patient. The level of immunosuppression was quantified using a composite drug score which includes both the number and dosage of individual drugs as described before[8].

Generation of lentiviral pseudoviruses and quantification of neutralizing antibody capacity

Lentiviral pseudoviruses were generated by co-transfection of HEK293T cells using FuGene 6 (Promega) with plasmids individually encoding for the RSV Long F protein, the RSV Long G protein with a C-terminal 31 amino acid deletion, HIV-1 Gag/Pol, HIV-1 Tat, HIV-1 Rev, and luciferase with ZsGreen. Cell culture supernatants were replaced on the day after transfection with fresh high-glucose DMEM supplemented with 1 mM sodium pyruvate, 2 mM L-glutamine, 100 U/mL penicillin, 100 µg/mL streptomycin (all from Thermo Fisher), and 10% FBS (Sigma-Aldrich). Supernatants containing RSV pseudoviruses were harvested 48 h after transfection,

clarified by centrifugation at 400 g and 4°C for 10 min, filtered using a 0.45 µm filter, and flash-frozen in a dry ice-ethanol bath.

To determine RSV neutralizing plasma activity, $2x10^4$ HEK293T cells per well were plated on a 96-well plate and cultured over night. On the next day, RSV pseudovirus-containing supernatants were co-incubated with a 1:3 dilution series of heat-inactivated (56°C for 30 min) heparinized plasma starting at a 1:200 plasma dilution. After coincubation for 60 min at 37°C and 5% CO_2, plasma/pseudovirus mixtures were added to the plated 293T cells and incubated for 48 h at 37°C and 5% CO_2. Subsequently, cells were lysed by addition of luciferin/lysis buffer (10 mM $MgCl_2$, 0.3 mM ATP, 0.5 mM coenzyme A, 17 mM IGEPAL CA-630 (all Sigma-Aldrich), and 1 mM D-Luciferin (GoldBio) in Tris-HCl). Lysates were transferred to a white 96-well plate and bioluminescence was determined using a microplate reader (Berthold). Fifty-percent plasma inhibitory dilutions (ID_{50}) were determined as the dilutions resulting in a 50% reduction of the above-background luminescence signal (determined using uninfected cells) relative to RSV pseudovirus-infected control cells using a four-parameter variable slope regression curve. All samples were tested in technical duplicates. Samples below or above the minimum or maximum ID_{50} were set at the minimum or maximum value of 200 and 437400, respectively.

Supplementary Figures

Supplementary Figure S1

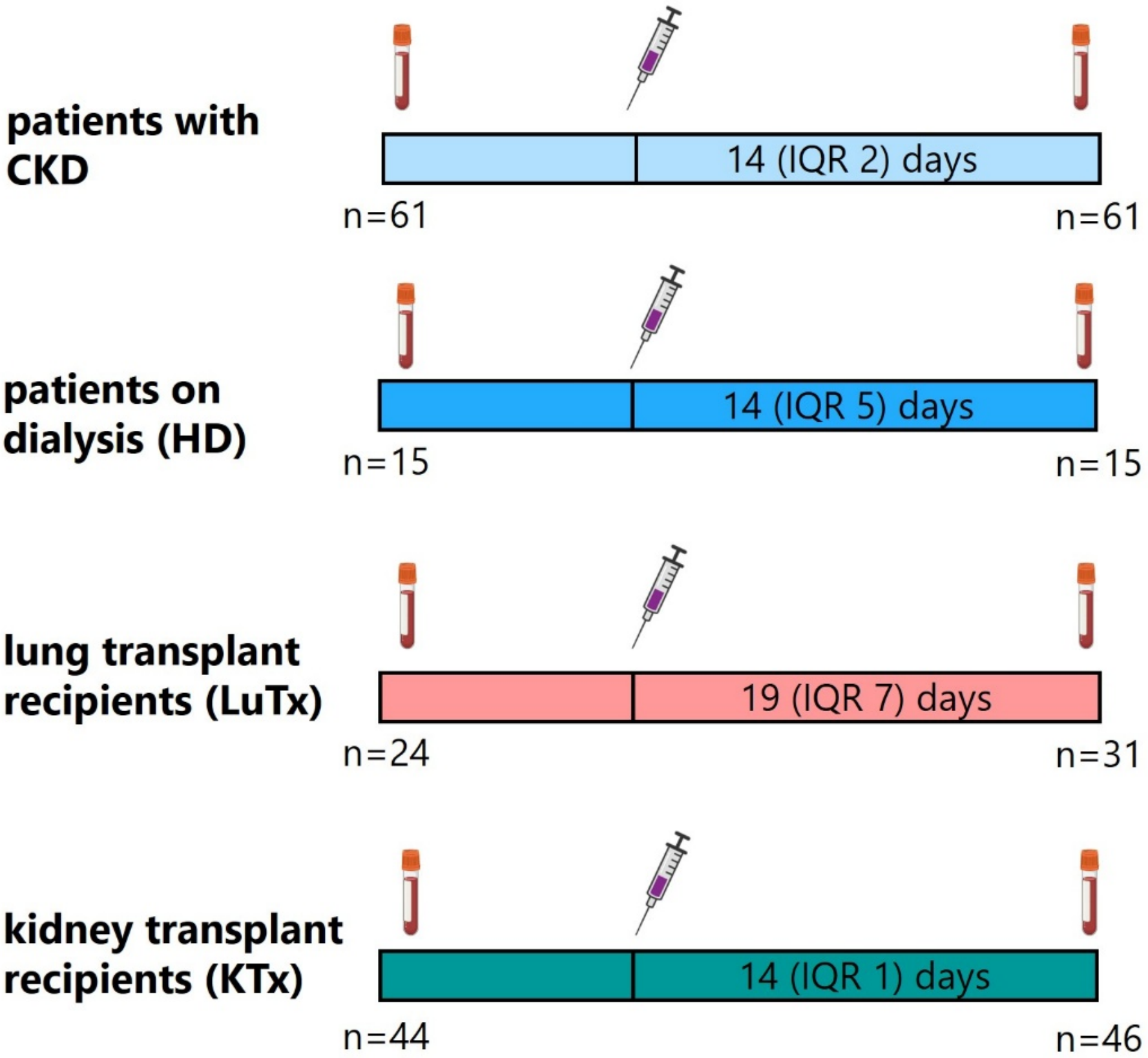

Supplementary Figure S1: Schematic overview of the study design and patient groups. Blood samples were drawn from patients with chronic kidney diseases (CKD), patients on intermittent maintenance hemodialysis (HD), kidney (KTx) and lung transplant (LuTx) recipients before and after vaccination with either the protein-based adjuvanted vaccine RSVPreF3-AS01$_E$ ("Arexvy", GSK) or the nonadjuvanted RSVpreF ("Abrysvo", Pfizer). One LuTx recipient had an RSV infection between vaccination and sampling after vaccination. This patient is shown in the display items but excluded from statistical analyses. Abbreviations: CKD, chronic kidney disease; IQR, interquartile range; KTx, kidney transplant; LuTx, lung transplant; preF, prefusion F; RSV, respiratory syncytial virus.

Supplementary Figure S2

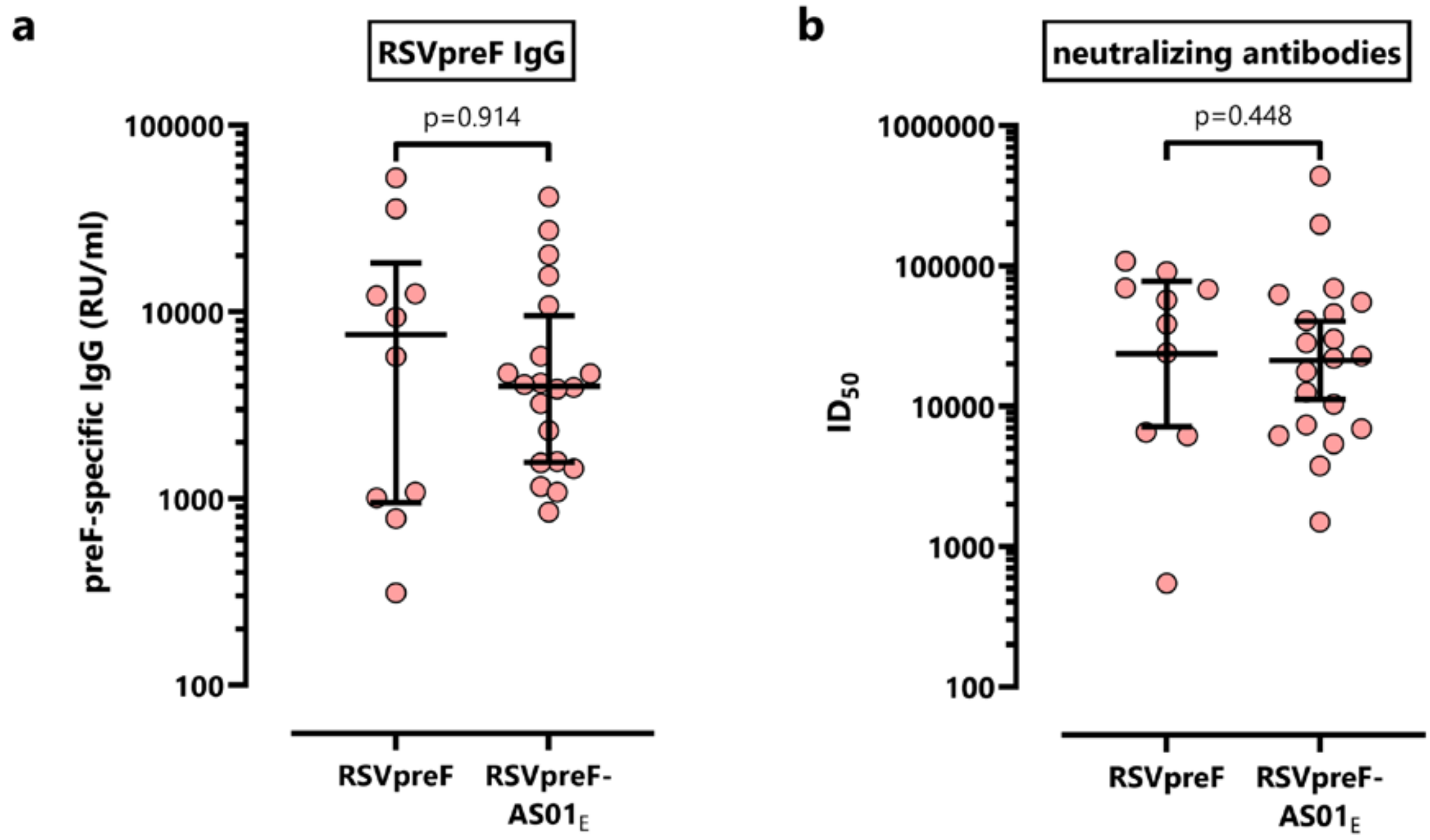

Supplementary Figure S2: Comparison of vaccine-induced humoral immunity between lung transplant recipients receiving either the adjuvanted RSVpreF3-AS01$_E$ or the non-adjuvanted RSVpreF vaccine. Levels of **(a)** preF-specific IgG or **(b)** neutralizing antibody activity were compared between lung transplant recipients vaccinated with either RSVpreF (Abrysvo, n=10) or RSVpreF3-AS01$_E$ (Arexvy; preF-specific IgG, n=19; nAb, n=20). Bars represent median titers with interquartile ranges for preF-specific IgG and fold increases, and geometric mean ID$_{50}$ with 95% CI for neutralizing antibodies. Differences between the groups were determined using the Mann-Whitney test. Abbreviations: AS01$_E$, adjuvant system 01$_E$; ID$_{50}$, half-maximal inhibitory concentration; Ig, immunoglobulin; preF, prefusion F; RSV, respiratory syncytial virus; RU, relative Unit.

Supplementary Tables

Supplementary Table S1: Demographic and clinical characteristics of the study population

		CKD[e]	HD	KTx	LuTx	p-value
		n=61	n=15	n=46	n=31	
Years of age, mean (SD)		73.8 (7.2)	76.8 (6.6)	67.8 (5.9)	60.9 (10.3)	<0.0001[a]
Sex, n (%)[f]						
	female	26 (42.6)	7 (46.7)	16 (34.8)	18 (58.1)	0.244[b]
	male	35 (57.4)	8 (53.3)	30 (65.2)	13 (41.9)	
RSV vaccine type, n (%)						
	RSVpreF3-AS01$_E$	61 (100)	15 (100)	45 (97.8)	21 (67.7)	
	RSVpreF			1 (2.2)	10 (32.3)	
Days since RSV vaccination, median (IQR)		14 (2)	14 (5)	14 (1)	19 (7)	0.004[c]
Years since transplantation, median (IQR)				5.9 (9.9)	5.3 (7.5)	0.701[d]
Patients within first year of transplantation, n (%)				8 (17.4)	5 (16.1)	
Immunosuppressive therapy		10 (16.4)[g]	1 (6.7)[g]	46 (100)	31 (100)	
Immunosuppressive regimen, n (%)[h]						
	triple			35 (76.1)	27 (87.1)	
	double			10 (21.7)	4 (12.9)	
	single			1 (2.2)	0 (0)	
MMF/MPA, n (%)						
	yes			40 (87.0)	17 (54.8)	
	no			6 (13.0)	14 (45.2)	
MMF or MPA dosage, n (%)						
	0 (none)			6 (13.0)	14 (45.16)	
	<1000 mg			14 (30.4)	3 (9.68)	
	1000 - <2000 mg			24 (52.2)	14 (45.16)	
	≥2000 mg			2 (4.4)	0 (0)	
Rituximab, n (%)		3 (4.9)	0 (0)	1 (2.2)	0 (0)	

[a]Ordinary one-way ANOVA, [b]Fisher's exact test, [c]Kruskal-Wallis test with Dunn's post test, [d]Mann-Whitney U test, [e]among patients with CKD, one was G2a, 18 were G3a, 21 were G3b, 17 were G4a, and 4 were G5a; [f]information on sex was based on individual self-declaration; [g]patients were currently undergoing treatment with calcineurin inhibitors, antimetabolites, glucocorticoids or complement inhibitors and/or receive B-cell or plasma cell-depleting therapy within the past 6 months. [h]triple: drugs including GC, antimetabolite and CNI or mTOR-I; double: GC in combination with CNI, antimetabolite or mTOR-I or CNI and antimetabolite; single CNI. One LuTx recipient had an RSV infection between vaccination and sampling after vaccination. Abbreviations: antimetabolite, azathioprine or mycophenolate mofetil/mycophenolic acid; AS01$_E$, adjuvant system 01$_E$; CKD, chronic kidney disease; CNI, calcineurin inhibitor; GC, glucocorticoids; HD, hemodialysis patients; IQR, interquartile range; KTx, kidney transplant recipients; LuTx, lung transplant recipient; mTOR-I, m-TOR inhibitor; preF, prefusion F; RSV, respiratory syncytial virus; SD, standard deviation.

Supplementary Table S2: Levels and quantitative changes in vaccine-induced preF-specific IgG antibodies and neutralizing antibody activity.

	CKD	HD	KTx	LuTx	p-value
**preF-specific IgG** (RU/ml)					
**pre** median (IQR)	576.9 (IQR 773.8)	488.4 (IQR 1286.7)	504.1 (IQR 552.8)	860.3 (IQR 845.7)	0.198
**post** median (IQR)	7754 (IQR 12289)	5863 (IQR 7179)	1843 (IQR 5552.3)	4114 (IQR 10910)	**<0.0001**
**fold change**, median (IQR)	11.9 (IQR 20.9)	7.8 (IQR 14.7)	3.3 (IQR 8.2)	7.6 (IQR 23.1)	**0.0002**
**neutralizing capacity** (ID_{50})					
**pre** geometric mean (95% CI)	2340 (95% CI 1623)	1273 (95% CI 665.6)	2498 (95% CI 1710)	3608 (95% CI 2437)	0.074
**post** geometric mean (95% CI)	31953 (95% CI 21392)	14488 (95% CI 5899)	10497 (95% CI 6003)	21983 (95% CI 12810)	**0.009**
**fold change**, median (IQR)	10.9 (IQR 32.9)	5.4 (IQR 49.0)	2.6 IQR 12.1)	7.5 (IQR 18.7)	**0.002**

Values are derived from patients where paired samples were available before and after vaccination.

Supplementary Table S3: Multivariate regression analyses of RSVpreF-specific IgG and neutralizing antibody activity between the four patient groups.

Dependent variables		IgG RSVpreF		neutralizing antibody activity	
		**Estimate (95% CI)**	**p-value**	**Estimate (95% CI)**	**p-value**
**Group**	KTx (ref)	1		1	
	LuTx	0.290 (0.009 - 0.571)	**0.043**	0.229 (-0.125 - 0.584)	0.204
	CKD	0.599 (0.368 - 0.830)	**<0.0001**	0.535 (0.243 - 0.827)	**0.0004**
	HD	0.507 (0.157 - 0.857)	**0.0048**	0.218 (-0.226 - 0.662)	0.334
**Age**		-0.007 (-0.020 - 0.005)	0.250	-0.010 (-0.025 - 0.005)	0.203
**Sex**	male (ref)	1		1	
	female	0.083 (-0.103 - 0.270)	0.379	0.100 (-0.136 - 0.335)	0.404

Shown are p-values of multivariate linear regression analyses with log(10) transformed values after vaccination; parameters refer to RSV-specific humoral immune responses (preF-specific IgG and neutralizing activity) after vaccination with KTx recipients and male individuals as a reference for categorical parameters. One LuTx recipient had an RSV infection between vaccination and sampling after vaccination and was therefore excluded from statistical analysis. Abbreviations: CI, confidence interval; CKD, chronic kidney disease; HD, hemodialysis; Ig, Immunoglobulin: KTx, kidney transplant recipients; LuTx, lung transplant recipients; preF, prefusion F; nAb, neutralizing antibodies; RSV, respiratory syncytial virus.

Supplementary Table S4: Median changes between vaccine-induced humoral and CD4 T cellular immune parameters.

	CKD	HD	KTx	LuTx	p-value
**preF-specific IgG**	11.9 (IQR 20.9) n=59	7.8 (IQR 14.7) n=15	3.3 (IQR 8.2) n=43	7.6 (IQR 23.1) n=23	**0.0002**
**neutralizing antibody activity**	10.9 (IQR 32.9) n=61	5.4 (IQR 49.0) n=15	2.6 (IQR 12.1) n=43	7.5 (IQR 18.7) n=23	**0.002**
**pan-RSV-specific IgG**	1.2 (IQR 0.4)* n=61	1.1 (IQR 0.1)* n=14	1.2 (IQR 0.7)# n=43	1.2 (IQR 0.4)# n=22	0.161
**F-specific IgG**	2.8 (IQR 7.0) n=60	2.1 (IQR 11.5) n=14	1.3 (IQR 0.7)# n=43	1.4 (IQR 4.0)# n=21	**0.0015**
**RSV-specific CD4 T cells**	4.1 (IQR 2.2)* n=58	4.4 (IQR 3.1)* n=12	4.6 (IQR 11.4)# n=43	2.1 (IQR 2.1)# n=22	**0.036**

Median (IQR) fold changes are derived from patients where paired samples were available before and after vaccination; one LuTx recipient had an RSV infection between vaccination and sampling after vaccination and was therefore excluded from statistical analysis. Compiled data on pan-RSV-specific IgG, F-specific IgG and RSV-specific CD4 T cells shown in the lower part of the table and are derived in part from[2] (labelled with *) and from[1] (labelled with #). Abbreviations: CKD, chronic kidney disease; HD, patients on hemodialysis; Ig, Immunoglobulin: KTx, kidney transplant recipients; LuTx, lung transplant recipients; preF, prefusion F; RSV, respiratory syncytial virus.

Supplementary References

1. Bronder S, Abu-Omar A, Lennartz S, et al. Cellular and humoral immunogenicity of respiratory syncytial virus vaccination in solid organ transplant recipients. *Am J Transplant.* 2026;26(3): 499-511.

2. Radun R, Bronder S, Abu-Omar A, Fliser D, Sester M, Schmit D. Immunogenicity and reactogenicity of the adjuvanted respiratory syncytial virus vaccine in patients with chronic kidney disease. *Clin Kidney J.* 2026;19(5): sfag093.

3. Falman A, Schönfeld V, Flasche S, et al. Beschluss und Wissenschaftliche Begründung zur Empfehlung der STIKO für eine Standardimpfung gegen Erkrankungen durch Respiratorische Synzytial-Viren (RSV) für Personen ≥ 75 Jahre sowie zur Indikationsimpfung von Personen im Alter von 60 bis 74 Jahren mit Risikofaktoren. *Epid Bull* 2024;32: 3-28.

4. Sester U, Schmidt T, Kuhlmann MK, Gartner BC, Uhlmann-Schiffler H, Sester M. Serial influenza-vaccination reveals impaired maintenance of specific T-cell memory in patients with end-stage renal failure. *Vaccine.* 2013;31(38): 4111-4120.

5. Bronder S, Mihm J, Urschel R, et al. Potent induction of humoral and cellular immunity after bivalent BA.4/5 mRNA vaccination in dialysis patients. *NPJ Vaccines.* 2024;9(1): 25.

6. Schmidt T, Klemis V, Schub D, et al. Immunogenicity and reactogenicity of heterologous ChAdOx1 nCoV-19/mRNA vaccination. *Nat Med.* 2021;27(9): 1530-1535.

7. Urschel R, Bronder S, Klemis V, et al. SARS-CoV-2-specific cellular and humoral immunity after bivalent BA.4/5 COVID-19-vaccination in previously infected and non-infected individuals. *Nat Commun.* 2024;15(1): 3077.

8. Sester M, van Leth F, Bruchfeld J, et al. Risk assessment of tuberculosis in immunocompromised patients. A TBNET study. *Am J Respir Crit Care Med.* 2014;190(10): 1168-1176.
